# Cytophagic histiocytic panniculitis: is it a macrophage activation syndrome in situ?

**DOI:** 10.1186/1546-0096-9-S1-P308

**Published:** 2011-09-14

**Authors:** B Bader-Meunier, S Fraitag, CEI Janssen, G de Saint Basile, K Brochard, N Brousse, C Bodemer, C Wouters

**Affiliations:** 1 Department of Pediatrics, Paris, France; 2 Department of Pathology, Paris, France; 3 Department of Pathology, Leuven, Belgium; 4 INSERM U 768, Paris France; 5 Department of Pediatrics, Toulouse, France; 6 Department of Dermatology, Paris, France; 7 Department of Pediatrics, Leuven, Belgium

## Background

Cytophagic histiocytic panniculitis (CHP) is a rare lobular panniculitis, characterized by subcutaneous proliferation of benign-appearing cytophagic histiocytes and lymphocytes often associated with systemic macrophage activation syndrome (MAS). Its pathogenesis is unknown.

## Aim

To report on immunohistochemical findings on skin CHP tissue.

## Patients

A 10-month-old year boy and a 4-month-old-year girl presented with multiple erythematous and indurated noduless on the limbs, and developed high-grade fever and biological features suggestive of MAS several months after the onset of cutaneous disease. Serologic test for EBV was negative and bone marrow examination revealed hemophagocytosis. A homozygous splicing mutation for the UNC13D gene encoding for Munc 13-4 was present in patient 1, but NK cell cytotoxicity and degranulation assays, and expression of Munc 13-4 protein were normal. No mutation was found in Patient 2. Prednisone alone failed to induce a sustained remission which was obtained by adding cyclosporine A.

## Results

Skin biopsy specimen revealed a lobular panniculitis with areas of necrosis in both patients. The inflammatory infiltrate consisted of lymphocytes and macrophages in dermis and hypodermis, and signs of hemophagocytosis. There were no atypical cells. These findings were consistent with CHP. Immunohistochemical stainings showed numerous CD68 macrophages and CD8+lymphocytes, expressing HLA-DR, and a few CD4+ cells. Lymphocytes expressed IFN-γ and macrophages TNF-α and IL6. These immunohistochemical features were characteristic of MAS.

## Conclusion

This study provides evidence that CHP may represent MAS *in situ* in adipose tissue; and emphasized that CHP can precede systemic signs of MAS by several months.

